# Susceptibility of *Legionella* Strains to the Chlorinated Biocide, Monochloramine

**DOI:** 10.1264/jsme2.ME12205

**Published:** 2013-09-03

**Authors:** Delphine Jakubek, Carole Guillaume, Marie Binet, Gérard Leblon, Michael DuBow, Matthieu Le Brun

**Affiliations:** 1 EDF R&D, Département LNHE, 6 quai Watier, 78400 Chatou, France; 2 Univ Paris-Sud, Institut de Génétique et de Microbiologie, CNRS UMR 8621, Bâtiment 409, 91405 Orsay cedex, France; 3 Euro Engineering, Énergie-Environnement, 22 terrasse Bellini, 92800 Puteaux, France

**Keywords:** *Legionella*, water cooling circuit, monochloramine, disinfection, biocide

## Abstract

Members of the *Legionella* genus find suitable conditions for their growth and survival in nuclear power plant cooling circuits. To limit the proliferation of *Legionella* pathogenic bacteria in nuclear power plant cooling circuits, and ensure that levels remain below regulatory thresholds, monochloramine treatment can be used. Although the treatment is highly effective, *i.e.* it reduces *Legionella* numbers by over 99%, *Legionella* bacteria can still be detected at low concentrations and rapid re-colonisation of circuits can occur after the treatment has ceased. The aim of this study was to develop an *in vitro* methodology for determining the intrinsic susceptibility of *L. pneumophila* strains, collected from various nuclear power plant cooling circuits subjected to different treatment conditions. The methodology was developed by using an original approach based on response surface methodology (RSM) combined with a multifactorial experimental design. The susceptibility was evaluated by the Ct factor. The susceptibility of environmental strains varies widely and is, for some strains, greater than that of known tolerant species; however, strain susceptibility was not related to treatment conditions. Selection pressure induced by monochloramine use did not result in the selection of more tolerant *Legionella* strains and did not explain the detection of *Legionella* during treatment or the rapid re-colonisation of cooling circuits after disinfection has ceased.

*Legionella pneumophila* is the causative agent of Legionnaires’ disease and *L. pneumophila* serogroup 1 is responsible for more than 98% of legionellosis cases in France (9). Bacteria of the genus *Legionella* are hydrotelluric and are found in natural as well as in artificial aquatic environments. The most frequently identified sources of legionellosis cases are hot water system networks, air conditioning systems and cooling towers (9). Bacteria in nuclear power plant cooling circuits can find suitable conditions for their survival and growth. Although circuit design is not conducive to the development of *Legionella* (due to water velocity, little backwater, etc.), the presence of nutrients (from suspended solids, organic matter, etc.), favourable growth temperatures (30 to 50°C for hot parts and 18 to 38°C for cold parts), the presence of oxygen, and the presence of biofilms and protozoa can provide a suitable environment for the development of these bacteria. Even if the cooling towers in nuclear power plants in France have never been implicated in legionellosis cases, regulatory monitoring of *Legionella* concentrations in cooling circuit water was established in France in 2004. This involves counting culturable *Legionella* spp. and *L. pneumophila* using the French Standard methodology (3) followed by serogroup determination (1 or 2 to 14) by latex agglutination (16, 18). If the concentrations are above the regulatory thresholds, corrective actions, such as disinfection procedures, are required. Thus, in some cooling circuits, chemical treatment with monochloramine is used to limit *Legionella* proliferation and ensure that the concentration is maintained below the authorised thresholds.

Oxidising biocides are characterised by their non-selective attack on microorganisms and by a common chemical feature: production of hydroxyl radicals (^•^OH), which are able to oxidise and mineralise almost any organic molecule, yielding CO_2_ and inorganic ions (25). Killing by active chlorine compounds proceeds in three steps: (i) formation of a chlorine cover (*i.e.* covalent N-Cl bonds) on the surface of the microbes, which affects virulence but not viability; (ii) penetration through cell barriers and (iii) destruction of important cell components, such as proteins responsible for bacterial transport, respiration and substrate dehydrogenation (2, 20). The rate of penetration can vary to a large degree for the same agent, mainly depending on the cell wall structure of bacteria. Gram-negative bacteria have higher susceptibility to monochloramine than Gram-positive bacteria, as demonstrated by Arnitz *et al.* (2); however, the specific mode of action of monochloramine on bacterial cells is not well known. Laboratory studies have shown that monochloramine does not severely damage the cell envelope or affect nucleic acid function; it reacts rapidly with only four amino acids (cysteine, cystine, methionine and tryptophan) and very slowly with DNA or RNA (20). In addition to the reactivity of the oxidant, the physiological state of the bacteria can influence the efficiency of bacterial inactivation by the oxidant (27). Monochloramine treatment is effective in reducing *Legionella* colonisation in water systems (16), such as in nuclear power plant cooling circuits (unpublished data). In these systems, monochloramine treatment is able to reduce culturable *Legionella* concentrations to below the enumeration method’s detection limit (500 CFU L^−1^). However, during disinfection, *Legionella* bacteria can still be detected at low concentrations and rapid re-colonisation in cooling circuits, as in other man-made water systems, can be observed after treatment has ceased (12, 18).

The survival of *Legionella* bacteria in water systems during treatment could be linked, in part, to the presence of protozoa (1, 8, 31). Their interaction allows *Legionella* to persist for long periods of time in the presence of biocide (1, 31). Thomas *et al.* (31) suggested that amoebae act as reservoirs for *L. pneumophila* and allow the rapid re-colonisation of water systems once treatment is interrupted. Biofilms are suspected to be the primary source of microorganisms in drinking water distribution systems. It has been shown that disinfection with biocides, such as chlorine dioxide and chlorite, can reduce the concentrations of planktonic bacteria, but has little or no effect on the concentrations of biofilm bacteria (17). Cooper *et al.* (12) showed that *L. pneumophila* biofilms were able to survive for 28 days when exposed to chlorine treatment at a concentration of 50 mg L^−1^. Another study performed on a pilot-scale water distribution system found that monochloramine did not deter *L. pneumophila* from accumulating in biofilms (22). However, monochloramine, and its low reactivity with biofilm polymers, has a better penetration into biofilms than free chlorine and is therefore more effective in eradicating *Legionella* in biofilm (23). Other studies have suggested that disinfection efficacy could be affected by microbial community diversity and, in turn, that the disinfection strategy could influence microbial diversity (8). For example, Pryor *et al.* (26) performed a study on biofilms from a water distribution system and showed that the use of monochloramine induces a larger decrease in *Legionella* diversity than free chlorine, confirming the high efficiency of monochloramine against *Legionella* in biofilm. Another hypothesis that could explain the persistence of *Legionella* in cooling circuits during treatment is the selection, by monochloramine, of *Legionella* strains that are more tolerant to this biocide (18). Although the consensus view is that chlorinated biocide usage does not induce bacterial resistance, the selection of intrinsically-tolerant strains by monochloramine cannot be excluded. Thus, it is necessary to determine the intrinsic susceptibility of *Legionella* strains isolated from cooling circuits, and to compare the susceptibility of strains isolated from non-treated cooling circuits with those from treated systems.

To achieve this objective, an *in vitro* approach, based on the Chick and Watson model (11, 33), was developed to determine Ct_99.9%_ values, the product of monochloramine concentration and the contact time necessary to observe a 3-log reduction in bacterial concentration. Ct values are directly proportional to bacterial inactivation rates. They represent the susceptibility of bacteria to the biocide under defined experimental conditions. The inactivation rates of bacteria with biocide, and the Ct values, are influenced by experimental parameters. An original approach, using response surface methodology (RSM) combined with a multifactorial experimental design, which is a mathematical method for designing experiments, building models, evaluating the effects of variables and searching optimum conditions of variables to predict responses, was used to obtain the optimum inactivation conditions in terms of temperature, pH, initial bacteria and biocide concentrations. Treating each factor separately would be very time-consuming. Furthermore, if several factors were to play a role, their interactions would not be discernible even if they were dominant. Hence, the application of an adequate experimental design is the optimal strategy to obtain maximum information with a minimum number of experiments. Moreover, RSM can provide an empirical model of the disinfection kinetics, based on the diverse variables of interest.

With the aim of determining the intrinsic susceptibility of *Legionella* strains, the disinfection kinetics of monochloramine on *Legionella* bacteria isolated from various treated or non-treated cooling circuits was investigated. Their susceptibilities were compared with those of bacteria taken from reference collections (*Legionella* and non-*Legionella* strains).

## Materials and Methods

### Bacterial strains and culture conditions

A set of 39 *L. pneumophila* strains was used to evaluate their susceptibilities to monochloramine (Table 1). These strains originated from water or biofilms and were isolated from a river, upstream or downstream from a nuclear power plant, or directly from various cooling circuits in nuclear power plants located in France. These cooling circuits were either non-treated or treated with monochloramine. *L. pneumophila* strains from the treated circuits were isolated over the course of the treatment and beyond or between two disinfection stages.

To compare the susceptibility of *L. pneumophila* with that of other *Legionella* species, 14 strains of *Legionella* non-*pneumophila* from reference collections (the American Type Culture Collection, ATCC, and the French Pasteur Institute Collection, CIP) were used (see Table 1). Susceptibilities of bacteria belonging to the genus *Legionella* were also compared with those of other bacteria belonging to non-*Legionella* genera. These non-*Legionella* strains belonged to Gram-negative and Gram-positive groups and were used to screen a wide range of susceptibilities.

All the strains were precultured in the laboratory before their use in inactivation studies. To limit the variability in the physiological state of bacteria, the incubation time necessary to attain the stationary state was observed depending on species. *Legionella* spp. strains were cultured on BCYE media supplemented with l-cysteine, and *L. pneumophila* on GVPC (Oxoid Microbiology Products, Cambridge, England), for four days at 37±2°C. *Escherichia coli*, *Pseudomonas fluorescens* and *Corynebacterium glutamicum* were cultured on R2A, Cetrimide medium and blood agar, respectively (Oxoid Microbiology Products), at 30±2°C for two days. *Staphylococcus aureus* and *Lactobacillus brevis* were cultured on Baird Parker and MRS media (Oxoid Microbiology Products, Thermo Fisher Scientific, Waltham, Massachusetts, USA) respectively, for two days at 37±2°C. After culture, colonies were suspended in sterile phosphate buffer (100 mM, pH 7.5) before disinfection treatment. The concentration was adjusted by A_595_ measurement at 595 nm (one A_595nm_ unit = 10^9^ cells mL^−1^).

*Legionella* strains isolated from the environment were subjected to comprehensive identification. Their genera, species and serogroups were identified using culture methods and latex agglutination in accordance with the AFNOR Standard method (3). The *mip* gene was sequenced to confirm the species identification (28) and a molecular typing method, the Infrequent-Restriction-Site PCR (IRS-PCR), was used to discriminate among *L. pneumophila* sub-populations (21).

### Monochloramine disinfection assays

Monochloramine was prepared by combining a predetermined volume of sodium hypochlorite to ammonia solution with a chlorine to nitrogen mass ratio of 4.8 and pH 8.3. Stock solutions of monochloramine at 1 g L^−1^ were stored at 4°C. Monochloramine concentrations were determined at the beginning and end of each assay using the DPD (N,N’-diethyl-*p*-phenylenediamine) (HACH Company, Loveland, Colorado, USA) colorimetric method in accordance with the manufacturer’s procedures.

Disinfection assays were performed by inoculating 10^8^ to 10^11^ bacteria per liter and 0.7 to 1 ppm monochloramine in sterile phosphate buffer (100 mM, pH 7.5). Samples were incubated at a controlled temperature (25°C–35°C) and pH (7.5–8.5) and were continuously agitated by magnetic stirring. The survival of the bacteria was analysed after 0, 5, 10, 15, 20, 25, 30, 45 and 60 min of treatment. Longer treatment was performed (90 to 120 min) on less susceptible strains. Samples were then treated with sterile sodium thiosulfate (20 mg L^−1^) to quench the monochloramine residual. Ten-fold serial dilutions were plated on the appropriate medium. The detection limit of the culture was 10^4^ CFU L^−1^. Bacterial concentrations were determined after a five-day culture for non-*Legionella* bacteria and after a ten-day culture for *Legionella* bacteria at the appropriate temperature. Disinfection assays were performed in triplicate for non-*Legionella* bacteria and only once for *Legionella* bacteria as the coefficient of variation of the method was determined for this genus (19%). For each experiment, a disinfectant consumption control without microorganisms and a bacterial survival control without biocide were performed to evaluate the stability of the biocide and the natural survival of the bacteria.

Experimental parameters, including temperature, pH, initial biocide and bacterial concentrations, were determined using a factorial design experiment combined with the RSM.

### Ct determination

Ct values were determined according to the Chick and Watson expression (11, 33), $log\frac{N}{N_{0}}=-kC^{n}t$, where *N_0_* is the initialnumber of culturable cells, *N* is the number of culturable cells after time *t* of disinfection exposure, *k* is the rate constant for a specific microorganism and set of conditions, *C* is the disinfectant concentration and *n* is the coefficient of biocide activity depending on the type of biocide and experimental variables.

Microorganism susceptibility was quantified by Ct (in mg·min^−1^ L^−1^). As frequently used in the literature, Ct values were calculated in our study for 3-log inactivation (Ct_99.9%_) (2, 15). The time necessary to inactivate 99.9% (t_99.9%_) of the bacteria was calculated by linear regression of the curve $log\frac{N}{N_{0}}=-f(t)$. The Ct value was the mathematical product of t_99.9%_ and the initial concentration of monochloramine.

### Development and optimisation of the method using the multifactorial experimental design and RSM

A multifactorial experimental design, combined with an RSM, was used to validate the microorganism susceptibility determination method. Two criteria, also called responses of the multifactorial experimental design, were chosen: (i) a significant reduction of the bacterial concentration (3-log bacterial reduction minimum) in approximately 30 min (Y_1_=t_99.9%_=30 min) and (ii) to ensure a minimal effect of the experimental variables on the effectiveness of the monochloramine, *i.e.* Y_2_=n=1. Four factors affecting the two responses, which would be easily controllable in the laboratory, were selected: temperature (X_1_), pH (X_2_), initial monochloramine concentration (X_3_) and initial bacterial concentration (X_4_). The four process parameters were added at two levels: low (−1) and high (+1). The low and high levels were chosen based on knowledge of the physicochemical characteristics of cooling waters with regard to temperature and pH, and the ability to obtain a rapid and detectable decay for monochloramine and bacterial concentration (Table 2). The central values (zero level) chosen were: T°=30°C, pH=8.0, [NH_2_Cl]=0.85 ppm and [bacteria]=3×10^9^ cells L^−1^. To develop the regression equation, the test variables were coded according to the following equation: $Xi=\frac{x_{i}-{\bar{x}}_{i}}{\Delta x_{i}}(i=1,2,3,4)$ where *Xi* is the coded value for the independent variable, *x_i_* is the real value of the independent variable, *χ̄_l_* is the real value of the independent variable at the centre point and Δ*x_i_* is the value of the step change. The response variables were fitted using a first order model in order to correlate response variables to the independent variables. The general form of the equation is:

$$Y=b_{0}+\sum_{i=1}^{n}b_{i}X_{i}+\sum_{i,j=1}^{n}b_{ij}X_{i}X_{j}+\sum_{i,j,k=1}^{n}b_{ijk}X_{i}X_{j}X_{k}+b_{ijkl}X_{i}X_{j}X_{k}X_{l}$$

where *Y* refers to the measured response, *X_i_*, *X_j_*, *X_k_* and *X_l_* to the independent coded variables, *b_0_* to the offset term, *b_i_*, *b_j_*, *b_k_* and b_l_ to the linear effects and *b_ij_*, *b_ijk_* and *b_ijkl_* to the interaction terms, and n corresponds to the number of studied factors. The multifactorial experimental design for four independent variables, each at two levels, consisted of 16 experiments, which permitted the determination of the *b* terms. Two additional experiments enabled model validation (Table 2). For each experiment, Y_1_=t_99.9%_ was measured as described in the previous section and Y_2_=n was calculated from the t_99.9%_ measured by pair tests where only the monochloramine concentration varied as below:

$$n=\frac{ln(t_{b99.9\%})-ln(t_{a99.9\%})}{ln(C_{a})-ln(C_{b})}$$

After modelling the responses, the RSM used a graphical representation to visualise the relationship between the response and the experimental levels of each variable to deduce the optimum conditions. Three-dimensional graphs were generated for the pairwise combination of two factors, while the other two were maintained at the extreme level (−1 or +1). The combination of optimum values reported for each interaction allowed us to determine the optimal experimental values for the method.

To validate the defined protocol, a reproducibility study was performed by independently testing the reference *L. pneumophila* strain ATCC 33152 eleven times. Method reproducibility was high, as the coefficient of variation determining the method error was 19% (data not shown).

## Results

### Protocol development using the multifactorial experimental design and RSM

The multifactorial experimental design was used to determine the optimum conditions, including temperature (X_1_), pH (X_2_), monochloramine (X_3_) and bacterial (X_4_) concentrations, to observe a 3-log bacterial reduction in approximately 30 min (Y_1_) and to optimise monochloramine activity (Y_2_). Sixteen experiments (runs n° 1 to 16) were then performed using the reference *L. pneumophila* strain ATCC 33152 and responses were experimentally determined (Table 2). Models were constructed to evaluate the effects of the parameters on responses:

$$\begin{array}{l} Y_{1}=318-239X_{1}+253X_{2}+205X_{3}+263X_{4}-282X_{1}X_{2}-234X_{1}X_{3}\\ -210X_{1}X_{4}+240X_{2}X_{3}+218X_{2}X_{4}+196X_{3}X_{4}-201X_{1}X_{2}X_{3}\\ -250X_{1}X_{2}X_{4}-221X_{1}X_{3}X_{4}+228X_{2}X_{3}X_{4}-188X_{1}X_{2}X_{3}X_{4} \end{array}$$

$$\begin{array}{l} Y_{2}=-0.3945+1.5229X_{1}-2.0595X_{2}-2.7756\;10^{-17}\;X_{3}\\ -0.6979X_{4}+0.3779X_{1}X_{2}+2.7756\;10^{-17}\;X_{1}X_{3}\\ +0.8645X_{1}X_{4}-2.7756\;10^{-17}\;X_{2}X_{3}-1.4079X_{2}X_{4}\\ +2.7756\;10^{-17}\;X_{3}X_{4}+2.7756\;10^{-17}\;X_{1}X_{2}X_{3}\\ -0.4254X_{1}X_{2}X_{4}-2.7756\;10^{-17}\;X_{1}X_{3}X_{4}\\ +2.7756\;10^{-17}\;X_{2}X_{3}X_{4}-2.7756\;10^{-17}\;X_{1}X_{2}X_{3}X_{4} \end{array}$$

To ensure their predictions, these models were tested under various experimental conditions, as shown in Table 2 (runs 17 and 18). Responses Y_1_ and Y_2_ were defined according to developed models (predicted responses) and experimental results (measured responses). For experimental conditions 17 and 18, predicted responses Y_1_ were 25.71 and 58.21 min, respectively, while measured responses Y_1_ were 35.63 and 49.92 min, respectively. For the pair of conditions 17 and 18, the predicted response Y_2_ was 1.43, whereas the measured response was 1.51. The residuals between predictive and real response values were low (less than 10 min for Y_1_ and less than 0.1 for Y_2_), meaning that the models could be validated.

**Fig. 1:**
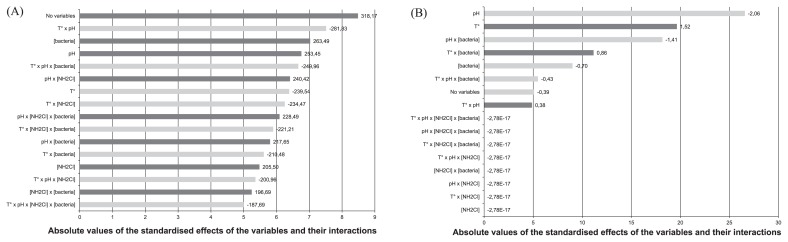
Bar graphs showing the standardised estimated effects of the variables tested against the time needed to inactivate 3-log units of bacteria (A) and the activity of monochloramine represented by the n factor (B) during disinfection assays with monochloramine. The variables tested were temperature, pH, initial monochloramine and bacterial concentrations. Standardised estimated effects correspond to the proportion of each estimated effect (absolute value) relative to the sum of all estimated effects.

The models developed in our study showed that all four variables, and their interactions, affect the contact time required to inactivate 99.9% of the bacteria (Fig. 1A), whereas only the temperature, pH and bacterial concentration, and their interactions, affect monochloramine efficiency (Fig. 1B). All experimental parameters had an impact on the t_99.9%_ with the same order of magnitude but, interestingly, the greatest effect on the response was not associated with any of the parameters tested, meaning that unmeasured experimental factors have a significant impact on the t_99.9%_. Temperature has a systematic negative effect on the response, whereas others parameters positively influence the t_99.9%_. Although almost all are equivalent, among the measured parameters, the combination of pH and temperature has the greatest influence on the time required to inactivate 3-log units of bacterial concentration. The efficiency of monochloramine is mostly influenced by pH and temperature, but in a converse manner, as pH affects monochloramine activity negatively and temperature affects it positively.

**Fig. 2:**
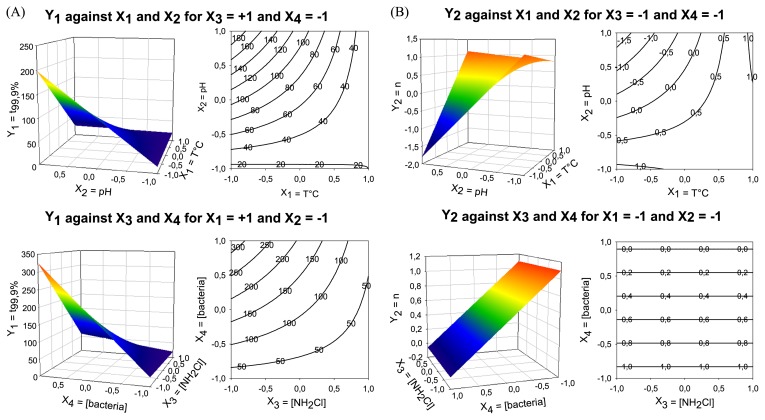
Response surface plots and contour plots of interactions between temperature and pH, while the other two variables (bacterial and biocide concentrations) are maintained at extreme levels, against the time necessary to inactivate 3-log units of bacteria, Y_1_=t_99.9%_.

RSM was applied to define the optimal conditions for monochloramine inactivation of bacteria. Optimum levels of temperature, pH, monochloramine and bacterial concentrations were determined by plotting response surface profiles against any two independent parameters, while keeping the other two at the extreme level (“−1” and “+1”). Thus, for one response, eight profiles were used within all possible combinations, to determine the optimal values of the four variables. Fig. 2 illustrates four profiles for the Y_1_ response surface plot in the optimisation of variables X_1_ and X_2._ For each profile, the optimal region was determined through visual inspection of the response surface plot. Optimal regions for Y_1_=30 min were combined to determine the solution interval of each variable. The solution intervals of each variable for Y_2_=1 were determined using the same methodology. The optimal values of temperature (X_1_), pH (X_2_), biocide (X_3_) and bacterial (X_4_) concentrations were then selected within the common interval of the two optimal regions computed for Y_1_=30 min and Y_2_=1. All of the curves used to optimise the variables are available as supplementary material. Indeed, the X_1_ level (temperature) needed to be between [−0.6; −0.2] or [0.8; +1], equivalent to [27; 29°C] or [34; 35°C]. The pH level (X_2_) was between 7.5 and 7.55. The initial concentration of monochloramine (X_3_) needed to be between 0.82 and 1.00 mg L^−1^. The initial bacterial concentration (X_4_) was between 2×10^8^ and 8×10^8^ CFU L^−1^. Our experimental conditions were then arbitrarily chosen from within the optimal intervals: T°=28°C, pH=7.5, [NH_2_Cl]_0_=0.9 mg L^−1^ and N_0_=5×10^8^ CFU L^−1^. The predicted and measured responses with these values were in agreement (data not shown).

### Susceptibilities of selected bacteria to monochloramine biocide

The aim of this study was to determine the susceptibilities of *Legionella* strains isolated from nuclear power plant cooling circuits under different disinfection conditions, and to compare these susceptibilities with those of reference strains, whether or not they belonged to the genus *Legionella*. The Ct_99.9%_ was measured, using the protocol defined by the multifactorial experimental design and the RSM, for non-*Legionella* bacteria and for *L. pneumophila* strains from the reference collections.

**Fig. 3:**
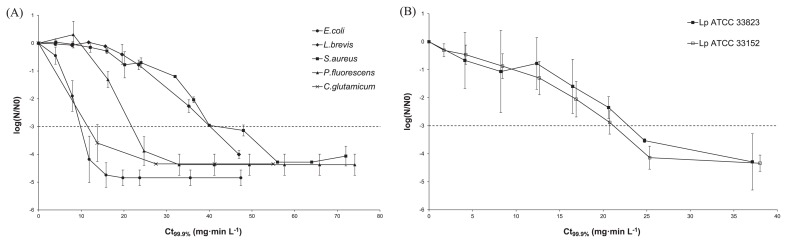
Reduction of non-*Legionella* bacteria (A) and *Legionella pneumophila* ATCC 33152 and ATCC 33823 (B) culturability after monochloramine treatments. Bars represent standard errors of the means of the three independent experiments.

Among the non-*Legionella* bacteria, *E. coli* was the most sensitive strain with a Ct_99.9%_ value of 10.3±1.67 mg·min L^−1^ followed by *C. glutamicum* (Ct_99.9%_=16.84±1.18 mg·min L^−1^), *P. fluorescens* (Ct_99.9%_=22.19±3.04 mg·min L^−1^), *L. brevis* (Ct_99.9%_=48.67±1.43 mg·min L^−1^) and *S. aureus*, which presented the lowest sensitivity with a Ct_99.9%_ value of 54.06±9.21 mg·min L^−1^ (Fig. 3A).

**Fig. 4:**
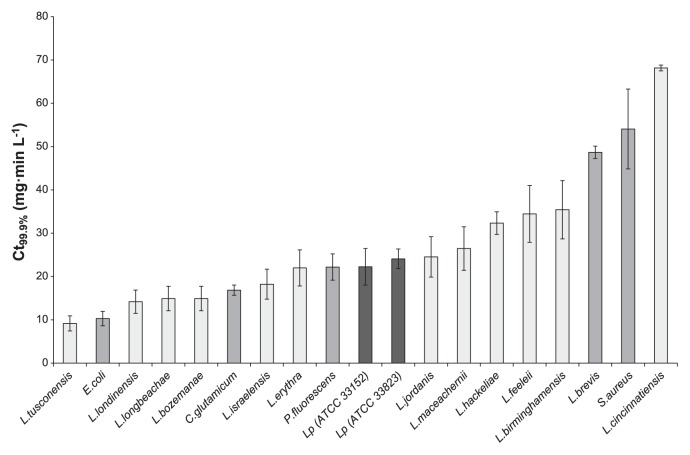
Ct_99.9%_ values after monochloramine treatment of non-*Legionella* and *Legionella* strains from the reference collections. Bars represent standard errors of the method (19%), except for non-*Legionella* bacteria and *L. hackeliae* and *L. cincinnatiensis*, for which bars represent standard errors of the mean of three independent experiments.

The two *L. pneumophila* reference strains, ATCC 33823 and ATCC 33152, showed the same inactivation kinetics and presented equivalent sensitivity against monochloramine (Fig. 3B). With Ct_99.9%_ values of 22.24±4.22 mg·min L^−1^ for the strain ATCC 33152 and 24.08±4.57 mg·min L^−1^ for strain ATCC 33823, the *L. pneumophila* species presented moderate sensitivity compared to other *Legionella* species and other non-*Legionella* strains (Fig. 4). Interestingly, the sensitivity of strains belonging to the genus *Legionella* extended to the widest range. Indeed, *L. tusconensis* was the most susceptible species (Ct_99.9%_=9.17±1.74 mg·min L^−1^) and was about seven times more susceptible than *L. cincinnatiensis* (Ct_99.9%_= 68.15±0.67 mg·min L^−1^).

**Fig. 5:**
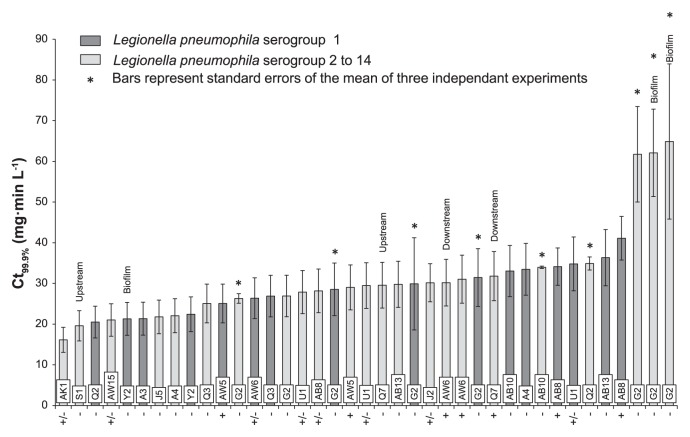
Ct_99.9%_ values after monochloramine treatment of environmental *Legionella pneumophila* strains isolated during various treatment conditions (− without treatment, + during treatment, +/− between two treatment phases) and identified by the IRS PCR method. Strains were collected from water or biofilm; upstream, inside or downstream from the cooling circuits. Tags represent the treatment condition during strains isolation.

The Ct_99.9%_ values of the environmental *L. pneumophila* strains ranged between 16.14±3.07 mg·min L^−1^ and 64.88±19.07 mg·min L^−1^ (Fig. 5). The susceptibilities of the environmental strains matched the susceptibilities of the non-*Legionella* bacteria, situated between the susceptibilities of *L. tusconensis* and *L. cincinnatiensis*. As shown in Fig. 5, a ranking of strains based on their Ct_99.9%_ values did not reveal characteristics that would be able to explain their susceptibilities. Indeed, it appeared that the susceptibilities of the environmental *L. pneumophila* strains were not linked to either their geographical origin (geographical location of the plant and their location upstream, inside or downstream from the plant) or to their initial matrix (water or biofilm) or serogroup identification (1 or 2 to 14). Moreover, the treatment phase (with, without or between two monochloramine treatment phases) did not have any impact on *Legionella* susceptibility, meaning that the use of monochloramine in the cooling circuit would not select monochloramine-tolerant strains.

Interestingly, Ct_99.9%_ values followed a normal distribution except for the three most tolerant strains. These three strains presented high Ct_99.9%_ values (61.74±11.73; 62.09±10.72 and 64.88±19.07 mg·min L^−1^) and were statistically more tolerant than the other environmental *L. pneumophila* strains (Grubbs test, α=0.05). Their susceptibilities were higher than those of the non-*Legionella* bacteria, *S. aureus* (54.06±9.21 mg·min L^−1^) and *L. brevis* (48.67±1.43 mg·min L^−1^), but lower than that of *L. cincinnatiensis* (68.15±0.67 mg·min L^−1^). Interestingly, these three strains belonged to IRS-PCR type G2 and were isolated from various matrices and power plants that were not treated with monochloramine biocide. These three strains were subject to SBT typing (28) and were not identical according to their sequence types (data not shown). Other strains belonging to the IRS-PCR type G2 were tested but they presented moderate Ct_99.9%_ values, between 26.31±1.18 and 31.46±7.08 mg·min L^−1^; indicating that tolerance to monochloramine is not a characteristic of the entire G2 type. Moreover, no other links between *L. pneumophila* identification (serogroups and IRS-PCR types) and their monochloramine susceptibilities were observed during this study.

## Discussion

This study was performed to define the intrinsic susceptibility of *L. pneumophila* strains isolated from cooling circuits during different disinfection processes, and to determine whether biocide usage in artificial systems could select biocide-tolerant *Legionella*. To define bacterial monochloramine susceptibility, an *in vitro* method to determine Ct_99.9%_ values was developed. Ct_99.9%_ values are defined as the mathematical product of the biocide concentration (mg L^−1^) and time (minutes) required to inactivate 3-log units of bacterial concentration. The Ct parameter reflects the natural susceptibility of bacteria to the biocide (11, 33), although it is highly sensitive to experimental conditions (29). To develop a robust and reproducible method, optimum laboratory conditions, in terms of the temperature, pH, monochloramine and bacterial concentrations, were established based on a multifactorial experimental design combined with RSM. The parameters were optimised to meet two method validation criteria: (i) to observe a 3-log unit decay of *Legionella* in approximately 30 minutes and (ii) to retain significant monochloramine activity. This original approach appears to be ideal for obtaining a maximum of information with a minimum number of experiments. The temperature, pH and initial bacterial concentration appeared to have a significant effect on *Legionella* susceptibility to monochloramine. The temperature and pH, when combined, had a greater effect on the inactivation speed than when observed individually. In both cases, the effects of these two parameters were the reverse. Increasing the temperature had a negative effect on the 99.9% inactivation time and a positive effect on monochloramine activity, whereas increasing the pH presented a positive effect on the 99.9% inactivation time and a negative effect on monochloramine activity. Although experimental condition effects are generally measured based on Ct values, these results were in agreement with those observed in past studies. Thus, studies on *Cryptosporidium parvum* inactivated with monochloramine, at a constant concentration, have shown that pH has a positive effect on Ct values, whereas temperature presents a negative effect (14, 29). Modelling of the experimental outcomes showed that the 99.9% inactivation time was dependent on other unmeasured parameters. The effect of these unknown factors seemed to be significant and should be studied more thoroughly in order to identify factors that could modulate monochloramine efficiency under laboratory conditions (physiological status of bacteria, free chlorine and other chloramine residuals).

The protocol defined by the multifactorial experimental design and RSM was used to determine the intrinsic susceptibility of *L. pneumophila* strains isolated from various nuclear power plant cooling circuits during different treatment processes. The Ct_99.9%_ values of these strains were compared with those of non-*Legionella* and *Legionella* species from the reference collections. For non-*Legionella* bacteria, monochloramine susceptibility was ordered as follows (from the most to the least susceptible strain): *E. coli* < *C. glutamicum* < *P. fluorescens* < *L. brevis* < *S. aureus*. Thus, except for *C. glutamicum*, it appeared that monochloramine susceptibility was linked to Gram stain characteristics. Gram-negative bacteria presented lower Ct_99.9%_ values than Gram-positive bacteria. This is consistent with previous studies, which have shown that Gram-negative bacteria are generally more susceptible than Gram-positive bacteria. This is a result of the better penetration of monochloramine in Gram-negative bacteria than in Gram-positive bacteria (2, 32). *C. glutamicum*, a Gram-positive bacterium, exhibited a Ct_99.9%_ value between those for Gram-negative bacteria. This bacterium belongs to the suborder *Corynebacterineae*, in which *Mycobacterium* and *Norcardia* genera are also present. These three genera are known to produce a particular and complex cell envelope, containing various lipid species, as well as mycolic acid residues covalently linked to arabinogalactan which, in turn, is linked to peptidoglycan (4). Interestingly, in the literature, studies of the effectiveness of monochloramine on other *Corynebacterineae* have revealed a strong inter-species variability of susceptibility as *M. avium* revealed high resistance to monochloramine, whereas *M. terrae* appeared very sensitive (7, 24, 30).

This inter-species variability of monochloramine susceptibility was also observed among *Legionella* species. Indeed, among selected strains from the reference collections, *L. tusconensis* was the most susceptible strain, whereas *L. cincinnatiensis* was the least susceptible. While all species of *Legionella* exhibited Ct_99.9%_ values within the same range as other Gram-negative bacteria, surprisingly, *L. cincinnatiensis* presented a Ct_99.9%_ value higher than that of the Gram-positive *S. aureus* strain.

*L. pneumophila* strains isolated from the environment also showed a high degree of variability in terms of their monochloramine susceptibilities. These strains were more susceptible than Gram-positive bacteria, except for three strains which were less susceptible than Gram-positive bacteria but more than *L. cincinnatiensis*. These three strains belonged to IRS-PCR type G2 but were not identical according to their SBT profiles. Other G2 strains showed moderate monochloramine susceptibilities, suggesting that the observed monochloramine tolerance might be a characteristic of a subgroup of the whole G2 type. Bacterial susceptibility and tolerance to monochloramine could be explained by different membrane compositions (13) or cell responses to biocide exposure (6). To investigate these hypotheses, first it would be useful to better characterise the mode of action of monochloramine on bacterial cells and to determine which sites in the cell are the most affected by the biocide. Membrane characterisation of susceptible and tolerant *L. pneumophila* strains could be very informative on biocide susceptibility. Secondly, a study investigating the cellular response of bacterial cells to the presence of monochloramine would allow a better understanding of the mechanisms involved in bacterial tolerance. Such a study could be performed by analysing and comparing the transcriptomic responses of susceptible and tolerant strains. Berry *et al.* (6) have defined, by performing a comparative transcriptomic analysis of the response of *E. coli* to monochloramine, a core set of genes responsible for increased tolerance to stresses, known as the “stressome”. Identifying and comparing the gene expression involved in bacterial tolerance between susceptible and non-susceptible strains would aid our understanding of susceptibility variations within the same bacterial species, as in the case of *L. pneumophila* species isolated from cooling circuits.

Although environmental *L. pneumophila* biocide susceptibility was found to cover a wide range of Ct_99.9%_ values, the results from this study suggest that monochloramine usage in nuclear power plant cooling circuits does not select more tolerant strains. Indeed, their susceptibilities were not ranked according to the treatment conditions during their isolation. These results are in agreement with those of Garcia *et al.* (18), who performed a long-term environmental monitoring study of *Legionella* persistence in chlorinated systems. The authors showed, by measuring the minimum inhibitory and bactericidal concentrations (MIC and MBC), that biocide usage in water systems does not increase the tolerance of *Legionella* strains.

Moreover, despite the higher tolerance of some strains, considering the concentration of monochloramine used during the cooling circuit disinfection process (0.25±0.05 mg L^−1^), under these conditions, the theoretical time required to inactivate 99.9% of *Legionella* is approximately four hours. Given that the minimum residence time of bacteria in cooling circuits is approximately six hours, this shows that the disinfection process used to eradicate *Legionella* bacteria in nuclear power plant cooling circuits is efficient.

Thus, the detection of *Legionella* bacteria during monochloramine treatment, and the rapid re-colonisation of nuclear power plant cooling circuits after a disinfection process, cannot be explained by the selection of strains that are naturally more biocide-tolerant. These phenomena could be explained by the presence of viable but not culturable *Legionella* in water systems, or the protection by biofilm location or by higher organisms (such as amoebae) (1, 31). *L. pneumophila* could persist in the VBNC state after biocide treatment (1, 5). This low metabolic activity state could be responsible for the failure to culture viable *L. pneumophila* from treated circuits. Under favourable conditions, VBNC bacteria can recover their culturability and their ability to grow in cooling circuits. Also, *Legionella* bacteria can be internalised into higher organisms, such as amoebae, wherein they are protected from the action of biocide (1, 31). *Legionella* hosts probably act as reservoirs for *L. pneumophila*, allowing rapid re-colonisation of the water system once the treatments are interrupted. Another possible explanation is protection based on biofilm location. Biofilms are known to reduce biocide efficiency by acting as a physical barrier to biocide penetration (12, 17, 22). The salting-out of biofilm bacteria in the water phase could explain the detection of *Legionella* during treatment and the rapid re-colonisation of cooling circuits. Moreover, post-amoebic and sessile *Legionella* exhibit a different phenotype than planktonic *Legionella*, enhancing their tolerance to biocide through the synthesis of proteins involved in oxidative stress (10, 19).

In conclusion, this study showed that monochloramine usage in nuclear power plant cooling circuits does not induce selection pressure leading to the persistence of tolerant *Legionella* bacteria. Although *Legionella* are sometimes still detectable at low concentrations during the treatment process, and although the cooling circuits are often rapidly re-colonised after treatment has ceased, disinfecting these water systems with monochloramine is effective and is not related to re-colonisation. The origin of these phenomena remains unclear and they may be caused by environmental factors such as biofilm location and protozoa protection.

## Supplementary Material



## Figures and Tables

**Table 1:** Strains selected (non-*Legionella* bacteria, *Legionella* species, and environmental *L pneumophila* strains) for the determination of their susceptibility to monochloramine.

*Strain*		Serogroup	IRS-PCR	Year of sampling	Location	Original matrix	NH_2_Cl
Non-*Legionella* bacteria							

*Escherichia coli*	ATCC 10536						
*Staphylococcus aureus*	ATCC 6538						
*Lactobacillus brevis*	CIP 103474						
*Corynebacterium glutamicum*	ATCC 13032						
Pseudomonas fluorescens*							

*Legionella* bacteria							

Reference strains							

*Legionella pneumophila*	ATCC 33152	7	L1				
*Legionella pneumophila*	ATCC 33823	1	R1				
*Legionella feeleii*	ATCC 35849		BJ1				
*Legionella longbeachae*	ATCC 33484	2	V1				
*Legionella jordanis*	ATCC 33623		AM1				
*Legionella birminghamensis*	ATCC 43702		AN1				
*Legionella hackeliae*	ATCC 35250		AC1				
*Legionella londiniensis*	ATCC 49505		AG1				
*Legionella erythra*	ATCC 35303		AR1				
*Legionella cincinnatiensis*	ATCC 43753		AP1				
*Legionella israelensis*	ATCC 43119		AO1				
*Legionella tusconensis*	ATCC 49180		AD1				
*Legionella maceachernii*	ATCC 35300		AE1				
*Legionella bozemanae*	ATCC 35545	2	N1				

Environmental *L. pneumophila* strains							

Qee 400	*Lp*	1	Q2	2005	Cooling circuit	Water	−
Qee 403	*Lp*	1	Q3	2005	Cooling circuit	Water	−
Qee 527	*Lp*	1	AB10	2005	Cooling circuit	Water	−
Qee 529	*Lp*	1	A4	2005	Cooling circuit	Water	−
Qee 531	*Lp*	2 to 14	A4	2005	Cooling circuit	Water	−
Qee 532	*Lp*	2 to 14	AB10	2005	Cooling circuit	Water	−
Qee 533	*Lp*	2 to 14	Q2	2005	Cooling circuit	Water	−
Qee 534	*Lp*	2 to 14	Q3	2005	Cooling circuit	Water	−
Qee 766	*Lp*	1	Y2	2008	Cooling circuit	Water	−
Qee 773	*Lp*	1	Y2	2008	Cooling circuit	Biofilm	−
Qee 1825	*Lp*	2 to 14	G2	2009	Cooling circuit	Biofilm	−
Qee 1837	*Lp*	2 to 14	G2	2009	Cooling circuit	Biofilm	−
Qee 1885	*Lp*	1	A3	2009	Cooling circuit	Water	−
Qee 2343	*Lp*	1	AB8	2009	Cooling circuit	Water	+
Qee 4195	*Lp*	2 to 14	G2	2009	Cooling circuit	Water	−
Qee 4595	*Lp*	2 to 14	J2	2005	Cooling circuit	Water	+/−
Qee 4596	*Lp*	1	AW6	2005	Cooling circuit	Water	+/−
Qee 5008	*Lp*	1	AB8	2006	Cooling circuit	Water	+
Qee 5354	*Lp*	2 to 14	J5	2007	Cooling circuit	Water	−
Qee 5869	*Lp*	2 to 14	G2	2010	Cooling circuit	Water	−
Qee 5874	*Lp*	1	G2	2010	Cooling circuit	Water	−
Qee 6048	*Lp*	1	AW5	2010	Cooling circuit	Water	+
Qee 6054	*Lp*	2 to 14	AW5	2010	Cooling circuit	Water	+
Qee 6750	*Lp*	1	G2	2010	Cooling circuit	Water	−
Qee 6905	*Lp*	2 to 14	AB13	2010	Cooling circuit	Water	−
Qee 6918	*Lp*	1	AB13	2010	Cooling circuit	Water	−
Qee 7591	*Lp*	2 to 14	U1	2010	Cooling circuit	Water	+/−
Qee 7592	*Lp*	2 to 14	AK1	2010	Cooling circuit	Water	+/−
Qee 7604	*Lp*	2 to 14	U1	2010	Cooling circuit	Water	+/−
Qee 7605	*Lp*	1	U1	2010	Cooling circuit	Water	+/−
Qee 7614	*Lp*	2 to 14	AW15	2010	Cooling circuit	Water	+/−
Qee 7615	*Lp*	2 to 14	AB8	2010	Cooling circuit	Water	+/−
Qee 7748	*Lp*	2 to 14	S1	2010	Upstream	Water	−
Qee 7830	*Lp*	2 to 14	AW6	2010	Downstream	Water	+
Qee 7831	*Lp*	2 to 14	AW6	2010	Cooling circuit	Water	+
Qee 7841	*Lp*	2 to 14	Q7	2010	Upstream	Water	−
Qee 7842	*Lp*	2 to 14	Q7	2010	Downstream	Water	+
Qee 10246	*Lp*	1	G2	2011	Cooling circuit	Water	−
Qee 10420	*Lp*	2 to 14	G2	2011	Cooling circuit	Water	−

− Strain isolated from a non-treated cooling circuit

+ Strain isolated from a cooling circuit treated continuously with monochloramine

+/− Strain isolated from a cooling circuit treated sequentially with monochloramine and between two treatment phases

* Environmental origin

**Table 2:** Multifactorial experimental design matrix and measured responses for optimisation of experimental conditions (temperature, pH, [NH_2_Cl], [bacteria]).

Run order	Experimental conditions								Measured responses	
	
	X_1_		X_2_		X_3_		X_4_		Y_1_	Y_2_
				
	Temperature °C		pH		[NH_2_Cl] ppm		[bacteria] t_0_ cells mL^−1^		t_99.9%_ min	n
1	+ 1	35	+ 1	8.5	+ 1	1	+ 1	10^11^	97.71	−2.22
2	+ 1	35	+ 1	8.5	+ 1	1	−1	10^8^	23.75	1.1134
3	+ 1	35	+ 1	8.5	−1	0.7	+ 1	10^11^	44.18	−2.22
4	+ 1	35	+ 1	8.5	−1	0.7	−1	10^8^	35.33	1.1134
5	+ 1	35	−1	7.5	+ 1	1	+ 1	10^11^	58.6	4.81
6	+ 1	35	−1	7.5	+ 1	1	−1	10^8^	18.6	0.81
7	+ 1	35	−1	7.5	−1	0.7	+ 1	10^11^	326.1	4.81
8	+ 1	35	−1	7.5	−1	0.7	−1	10^8^	24.8	0.81
9	−1	25	+ 1	8.5	+ 1	1	+ 1	10^11^	3750	−6.9
10	−1	25	+ 1	8.5	+ 1	1	−1	10^8^	198.67	−1.81
11	−1	25	+ 1	8.5	−1	0.7	+ 1	10^11^	319.15	−6.9
12	−1	25	+ 1	8.5	−1	0.7	−1	10^8^	104.16	−1.81
13	−1	25	−1	7.5	+ 1	1	+ 1	10^11^	29.12	−0.06
14	−1	25	−1	7.5	+ 1	1	−1	10^8^	12.94	1.1
15	−1	25	−1	7.5	−1	0.7	+ 1	10^11^	28.5	−0.06
16	−1	25	−1	7.5	−1	0.7	−1	10^8^	19.2	1.1

17	0	30	−1	7.5	+1	1	−0.33	10^9^	35.63	1.51
18	0	30	−1	7.5	−0.33	0.8	−0.33	10^9^	49.92	1.51
