# Supplementary material for: Susceptibility of Legionella Strains to the Chlorinated Biocide, Monochloramine
Source: Microbes Environ. 2013 Sep 3;28(3):336–45. doi: 10.1264/jsme2.ME12205 (PMC4070956; doi:10.1264/jsme2.ME12205)
Supplement: Supplementary file 1 [file 28_336_s1.pdf]

SUPPLEMENTAL MATERIAL

SUSCEPTIBILITY OF *LEGIONELLA* STRAINS TO THE CHLORINATED BIOCIDE, MONOCHLORAMINE

Delphine Jakubek[a,b*], Carole Guillaume[c], Marie Binet[a], Gérard Leblon[b], Michael DuBow[b] and Matthieu Le Brun[a]

[a]EDF R&D, Département LNHE, 6 quai Watier, 78400 Chatou, France

[b]Univ Paris-Sud, Institut de Génétique et de Microbiologie, CNRS UMR 8621, Bâtiment 409, 91405 Orsay cedex, France

[c]Euro Engineering, Énergie-Environnement, 22 terrasse Bellini, 92800 Puteaux, France

[*] Corresponding author:
EDF R&D, Laboratoire National Hydraulique et Environnement, 6 quai Watier, 78400 Chatou, France
Email: delphinejakubek@hotmail.fr
Tel: + (33)130878641
Fax: + (33)130877336

Fig. S1 Response surface plots (left) and contour plots (right) of interactions between two variables (temperature and pH or initial monochloramine and bacterial concentrations), while the other two variables are maintained at extreme levels, on the time necessary to inactivate 3-log units of bacteria, $Y_1 = t_{99.9\%}$ and on the activity of monochloramine, $Y_2 = n$.

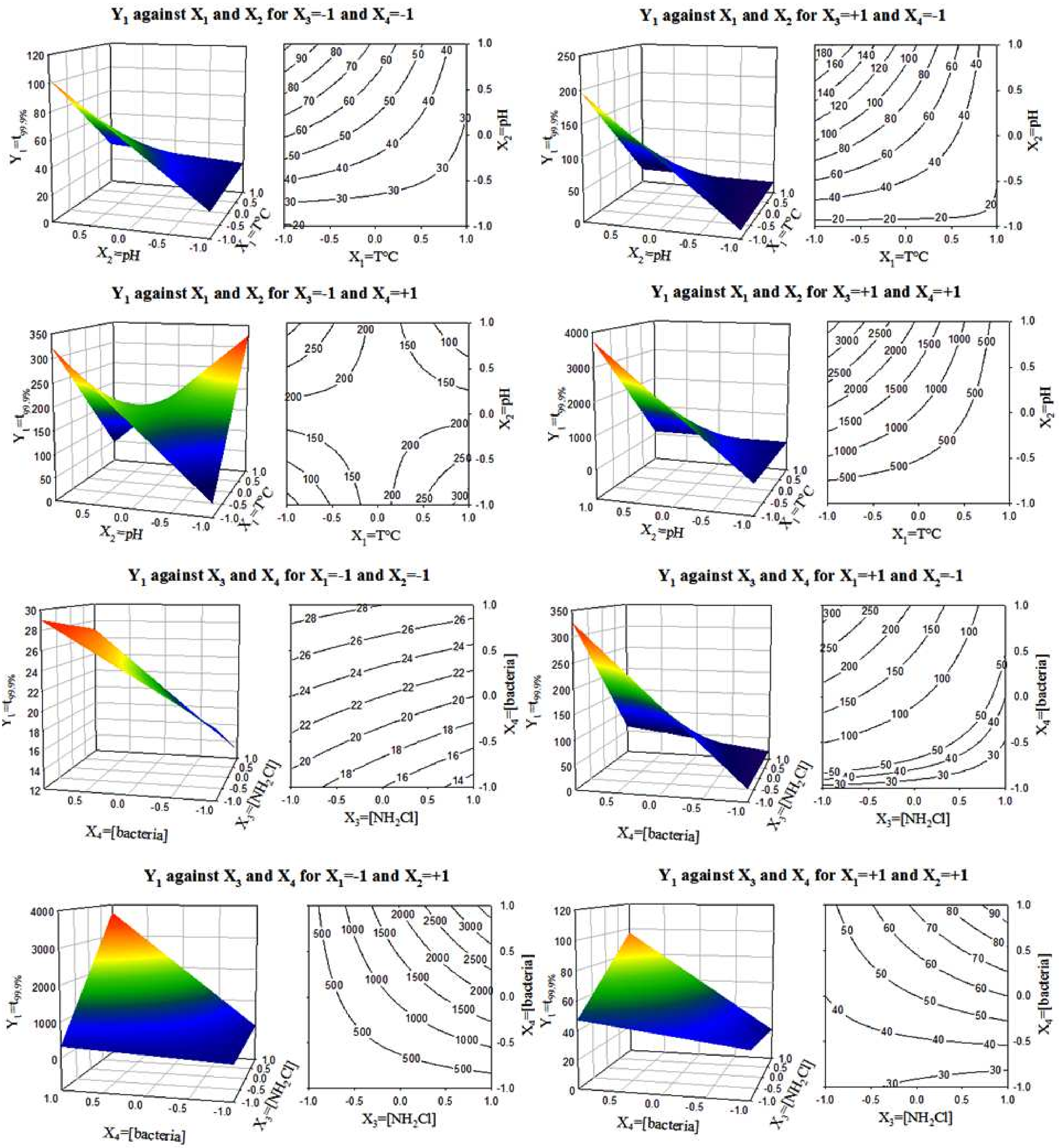

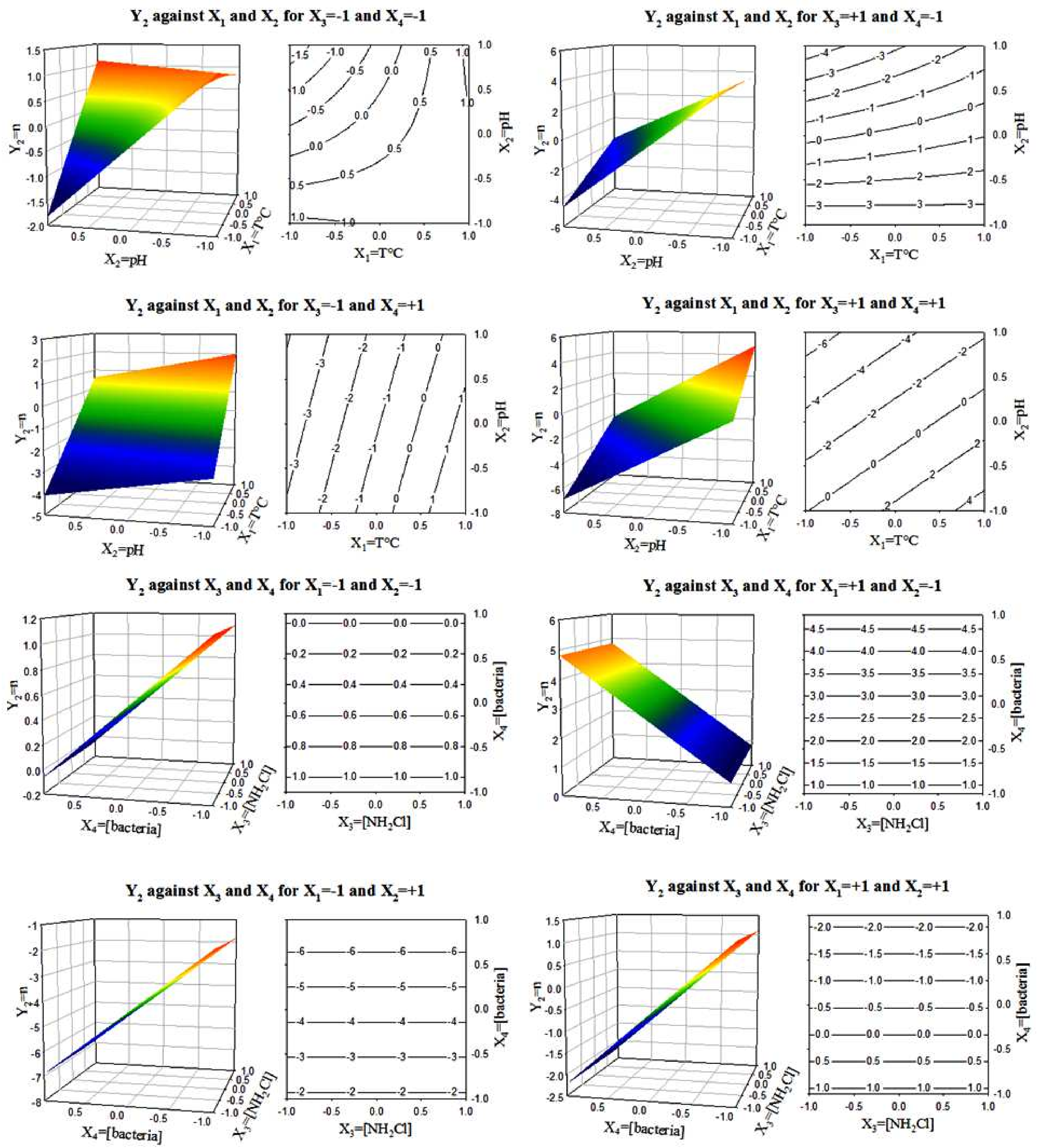

Y2 against X1 and X2 for X3=-1 and X4=-1
Y2 against X1 and X2 for X3=+1 and X4=-1
Y2 against X1 and X2 for X3=-1 and X4=+1
Y2 against X1 and X2 for X3=+1 and X4=+1
Y2 against X3 and X4 for X1=-1 and X2=-1
Y2 against X3 and X4 for X1=+1 and X2=-1
Y2 against X3 and X4 for X1=-1 and X2=+1
Y2 against X3 and X4 for X1=+1 and X2=+1
Y2=n
X1=T°C
X2=pH
X3=[NH2Cl]
X4=[bacteria]
